# Research on climate change based on carbonate porosity analysis in Jinping, China

**DOI:** 10.1371/journal.pone.0281630

**Published:** 2023-03-30

**Authors:** Honghai Kuang, Jinghao Li, Hailong Zuo, Xi Ye

**Affiliations:** 1 School of Geographic Science, Southwest University, Beibei, Chongqing, PR China; 2 School of Tourism and Environment, Zhangjiakou University, Zhangjiakou, Hebei, PR China; Northeastern University (Shenyang China), CHINA

## Abstract

In this paper, climate change in the Jinping area is investigated. The climate change trend in the Jinping area is studied by plotting the porosity value of the carbonate rocks as a curve. By comparing the curve established using the climate change data from published articles, it is found that the B value curve obtained using the saddle line is the closest to the curve established using the climate change data from published articles. This shows that the carbonate porosity in the Jinping area obtained using an image analysis technique can be used for climate change research.

## 1 Introduction

At present, many studies have been conducted on climate change. These studies started with loess, corals, tree rings, ice cores and stalagmites and effectively conducted climate change research. In areas where carbonate rocks are widely distributed, climate change studies cannot ignore the impact of karstification. The relationship between karst and climate is important. Under warm and humid climate conditions, the speed of karst development will be faster [1]. Therefore, the impact of karstification on climate change is an important topic in climate change research. The effects of karstification on climate change are commonly studied using stalagmites. However, the porosity of carbonate rocks is also an important karst research parameter, and it is also closely related to climate change. If the karst development rate of a carbonate formation increases, the porosity of the carbonate should also increase. If the karst development rate of a carbonate formation decreases, the porosity of the carbonate should also decrease. If the porosity of the carbonate rocks in the different strata in the Jinping area is used as the node of the curve to establish the porosity change curve, it should also reflect the climate change trend in the Jinping area. When it is also difficult to conduct climate change research on loess, sediments, and other materials locally, the carbonate porosity is a good parameter for studying climate change. The cost of collecting carbonate samples is lower than that of collecting stalagmites, and the processing and analysis costs are also lower than those for stalagmites. Therefore, the porosity of carbonate samples is a good tool for climate change research. If the porosity of carbonate rock samples is studied using the traditional carbonate rock research method (TCRM), it takes a long time. If climate change studies have to produce results within days, then the TCRM cannot be used. Image analysis methods are very suitable for obtaining climate change curves using porosity values over a short period of time.

Chinese scholars have attempted to study climate change through different methods. The warming and drying of the climate of China’s Loess Plateau and its impact on the ecological environment have been extensively studied [2]. The spatiotemporal dynamic characteristics of typical temperate glaciers in China have been examined [3]. Furthermore, the monsoon climatic record has been studied using oxygen and carbon isotope data from Porites lutea in the Shalao fringing reef, Hainan Island [4]. The response of the tree-ring width of Pinus sylvestris var. mongolica to climate change in the Hulunbuir Sandy Land has also been investigated [5]. In addition, the climate change trend on the Qinghai-Tibet Plateau over the past 30 years had been widely studied [6]. The geochemical record of terrestrial sediments in the Sea of Japan since the last ice age and its response to sea level and climate change has also been investigated [7]. These studies used a variety of methods to conduct climate change research, and they provide a good reference for the application of karst in climate change research. Runoff changes induced by vegetation recovery and climate change over carbonate and non-carbonate areas in the karst region of southwestern China has received increasing attention [8]. The spatial response of the ecosystem service value during the development of urban agglomerations has also been seriously studied [9]. Additionally, the thermal comfort of urban buildings based on local climate zones has been analyzed [10]. These studies revealed that carbonate rocks play a very important role in the study of climate change.

Chinese scholars have paid attention to the impact of climate change on human society. The optimization of local climate zones to mitigate urban heat island effect in human settlements has received much attention [11]. The contributions of urban ventilation to the thermal environment and the urban energy demand have been taken seriously [12]. The spatiotemporal relationship between the climate comfort of the urban human settlement environment and population density in China has also received much attention [13, 14]. These studies revealed that climate change is of great significance to human society.

Chinese scholars have attempted to use carbonate rocks to study climate change. The paleoclimatic change and termination of the last interglacial period using a stalagmite from Qingxin cave in south Guizhou has been investigated [15]. The vegetation, climate, and depositional environment changes since the Middle Holocene in the karst area of Guilin, Guangxi have also been studied [16, 17]. In addition, the discovery of glaciokarst in central Shandong Province and its climatic significance has been widely studied [18]. Moreover, the impacts of climate change on the vegetation in the Chongqing karst region has received much attention [19]. These studies have investigated the role of karst in climate change and are good references for this paper.

Many foreign scholars have paid attention to climate change research. A composite annual-resolution stalagmite record of the North Atlantic climate over the last three millennia has been studied [20]. Precise dating of the Dansgaard-Oeschger climate oscillations in western Europe using stalagmite data has been undertaken [21]. In addition, ancient mammalian and plant deoxyribonucleic acid (DNA) from Late Quaternary stalagmite layers in Solkota Cave has been analyzed [22]. The accurate dating of stalagmites with low seasonal contrast in the tropical Pacific climate using 2-D Sr maps, fabrics, and annual hydrological cycles has been studied [23]. The effects of changes in the Indian monsoon on Chinese stalagmite *δ*18O values during a simulated Heinrich event have received a great deal of attention [24]. These studies have demonstrated that carbonate rocks, represented by stalagmites, play a very important role in climate change research. These studies revealed that stalagmites are a good basis for climate change research. The research techniques of many other disciplines can be applied to climate change research based on stalagmite records. These studies also demonstrated that it is expensive to use stalagmites to study climate change; thus, it is necessary to develop reliable climate change research methods with a low research cost.

Except for stalagmites, most of the above studies on the role of karst in climate change research were conducted in very large research areas. In addition to stalagmites, these studies required many specific conditions for the research conducted, making reproducing these studies more difficult. As environmental protection laws become increasingly stringent, it is becoming more difficult to use stalagmites for climate change research. Compared with the difficulty of collecting carbonate specimens, the difficulty of collecting stalagmites is becoming increasingly difficult.

In some engineering constructions, owing to engineering safety requirements, it is necessary to understand the local climate change. Suitable stalagmites may not be available in the specific study area is of interest. The research methods used in the above studies on the role of karstification in climate change were not generally convinced by engineers. Owing to the time requirement of construction projects, the time allotted for the relevant paleoclimate research in the construction area is generally not very long. The Jinping area is such an area. In this area, there is a need for climate change research, and there is a wide distribution of carbonate rocks. There are few precedents for the study of loess and sediments in the Jinping area. Thus, it is not easy to use loess and sediment research for climate change research in the Jinping area. Therefore, reproducible, persuasive, and low-cost local climate change research methods are required. The large amount of carbonate rocks in the Jinping area provides a good foundation for this study.

Premise of this study is that during a warm and humid period, the karst development rate is fast, and the porosity of the carbonate rock is relatively large, whereas during a cold and dry period, the karst development rate is slow, and the porosity of the carbonate rock is relatively small. This is the same principle as that for microlayers in stalagmites. Therefore, the carbonate porosity, pore size, and other indicators provided by the carbonate rock polarized microscope images are good local climate change indicators. Obtaining the gray-scale curve for a polarized image of a carbonate rock is relatively simple [25]. However, the use of grayscale curves from polarized light microscopy images of carbonate rocks for climate change research requires comprehensive geoscientific analysis. The porosities of rock samples from carbonate formations are a good indicator for climate change studies. The climate change curve obtained using the porosity change curve of the rock sample will be easier to understand in the geological analysis. When rock samples are obtained from different formations, the time series can be determined. Since the porosity of each rock sample is directly affected by climate change, the porosities of the rock samples are a good parameter for climate change research. Therefore, in this study, the rock porosity was used to study the past climate change in the Jinping area. It should be noted that the changes in the porosities of the carbonate rocks only reflect climate change, and they do not directly reflect the temperature.

## 2 Materials and methods

### 2.1 Study site and samples

**Fig 1:**
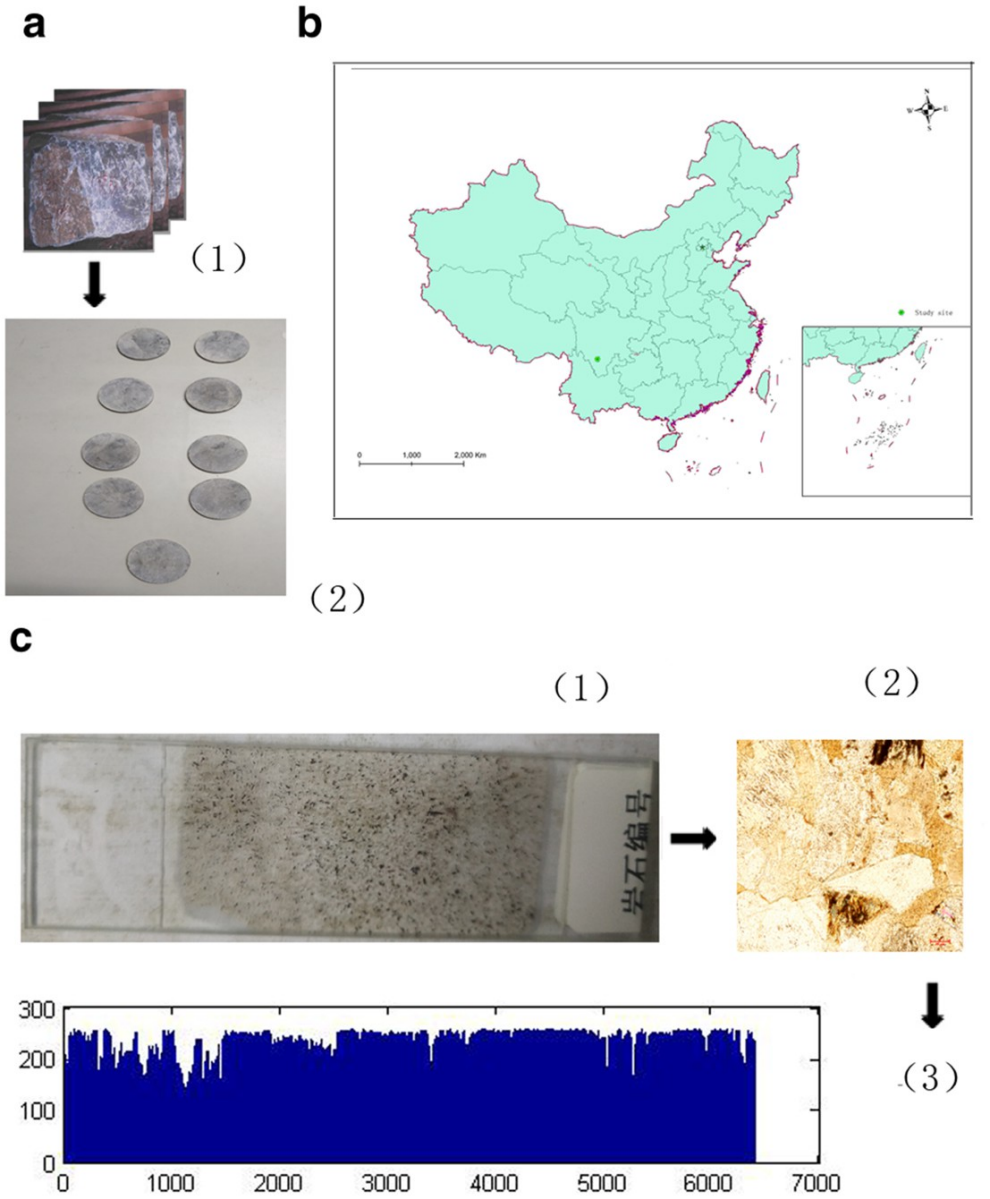
Study area and samples.

The Jinping area is a suitable area for studying the role of karstification in climate change research. There is a wide distribution of carbonate rocks in the area, and many carbonate rock specimens have been made into test pieces. Owing to the need for lithological analysis, many polarized light microscopy images of the carbonate rocks have been collected. Owing to the engineering construction, the local strata distribution is clear. Because of engineering construction reasons, the engineers in this area wish to know about the local climate changes. Therefore, the Jinping area is an ideal karst area for climate change research. In this study, nine rock samples from the Jinping area were selected and analyzed (Fig 1).

In Fig 1, a(1) shows a carbonate rock sample from Jinping: and a(2) is a carbonate rock specimen prepared from the sample shown in a(1) using the TCRM. Fig 1b shows a map of the Jinping area. Fig 1c(1) shows a rock slide made from the sample shown in Fig 1a(1).Fig 1c(2) shows a polarized image of the sample in Fig 1c(1) taken using a polarizing microscope. Fig 1c(3) shows the gray value curve for the sample shown in Fig 1c(2).

**Fig 2:**
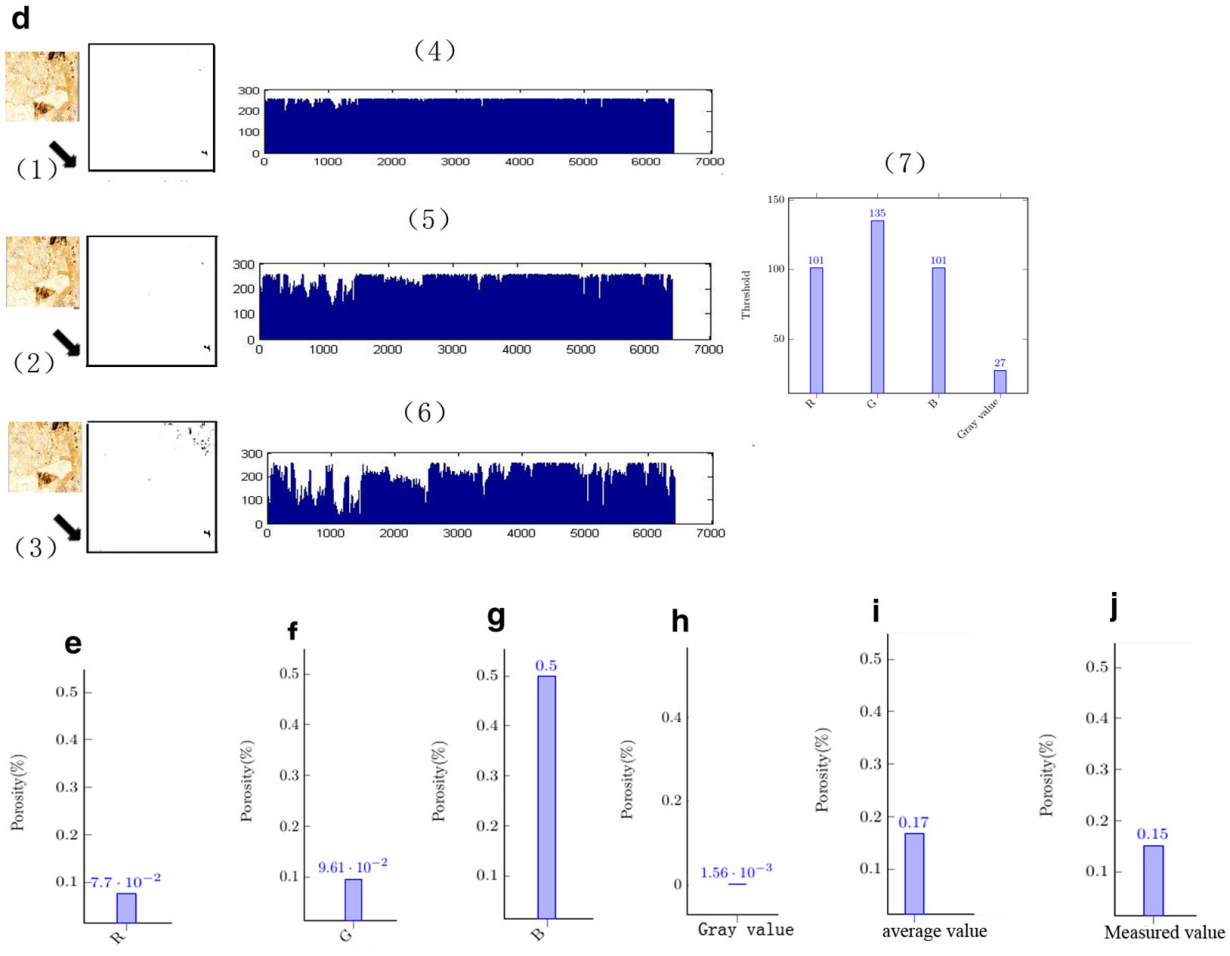
Comparison between carbonate image analysis and TCRM.

Whether carbonate rocks can be used to study climate change through image analysis of polarized light microscopy images depends on whether the polarized light microscopy images of carbonate rocks are suitable for research using image analysis technology. A comparison of the research results obtained using TCRM and the image analysis method can confirm whether the polarized microscopy images of carbonate rock are suitable for image analysis.Fig 2d(1) shows the conversion of the slide image into a binary image by using the R threshold to calculate the porosity.Fig 2d(4) presents the R value change curve. Fig 2d(2) shows the conversion of the slide image into a binary image by using the G threshold to calculate the porosity. Fig 2d(5) presents the G value change curve. Fig 2d(3) shows the conversion of the slide image into a binary image by using the B threshold to calculate the porosity. Fig 2d(6) presents the B value change curve. Through the analysis of these curves, it is easy to identify the threshold value of polarized microscopic images of carbonate rocks, as shown in Fig 2d(7). Fig 2d(7) shows the thresholds for the red-green-blue (RGB) values and grayscale values in Figs 1c(3), 2d(1)–2d(3). Figs 1c(3) and 2d(4)–2d(6) were used to analyze the distribution of the gray values and RGB values in the polarized light microscopic images of carbonate rock. Fig 2d(7) presents the threshold value for analyzing the gray value and RGB values of the carbonate rock polarized microscopic image in Figs 1c(3), 2d(4)–2d(6). Fig 2e shows the porosity of the carbonate slides obtained using the R value threshold in Fig 2d(7). Fig 2f shows the porosity of the carbonate slides obtained using the G value threshold in Fig 2d(7). Fig 2g shows the porosity of the carbonate slides obtained using the B value threshold in Fig 2d(7). Fig 2h shows the porosity of the carbonate slides obtained using the gray value threshold in Fig 2d(7). Fig 2i shows the average porosity of the carbonate slides obtained using the RGB value thresholds and the gray value threshold. If the local polarized light microscopic images of carbonate rocks are suitable for karst research using image analysis technology, the average value of the above mentioned thresholds (Fig 2i) and the measured value using TCRM should be similar. Fig 2j shows the porosity of the rock specimen corresponding to the carbonate rock slide measured using the TCRM method. The results shown in Fig 2i and 2j reveal that the average porosity obtained via image threshold analysis is very close to the porosity value measured using the TCRM method. This demonstrates that the image analysis method is a reliable method of obtaining the porosity of carbonate rocks.

Carbonate rocks are widely distributed in the Jinping area. These carbonate formations are distributed along *P*_1(*q*+*m*)_- *J*_1_. This constitutes an ideal time axis for climate change research in carbonate rock areas. The carbonate rocks in the Jinping area have experienced typical karstification. Porous carbonate rocks were formed in the Jinping area. The carbonate pores in these porous carbonate rocks provide a good research basis for climate change in the local carbonate rock area.

Climate change research requires a time axis, and the stratum is a good basis for constructing the time axis. In the carbonate rock area, if the collected carbonate rock samples include all of the carbonate rock strata, this is sufficient to establish the time axis for climate change research. If the research funds permit, it is suggested that more carbonate rock samples be collected from the same stratum, and the average porosity of these carbonate rock samples be taken as the basis for the climate change curve. This can improve the accuracy of the climate change curve.

### 2.2 Porosity analysis using a polarized light image of a carbonate rock and a finite automaton

When a polarized image of a carbonate rock is used to analyze the pores in the carbonate rock, the main method is to divide the number of pixels in the image that are pores by the total number of pixels. Thus, the premise of obtaining the porosity of the carbonate rock using the image analysis method is to correctly identify the carbonate pore pixels. In this study, in order to correctly identify the pore pixels in the polarized image of the carbonate rock, a finite automaton was used as the algorithm model for the carbonate rock image analysis. The gray value has been commonly used to conduct carbonate climate change research in previous studies, and the gray value formula is relatively easy to introduce in finite automata. Owing to the characteristics of finite automata, the modification to the mapping must be conducted using a finite number. The porosity change curve based on the gray value shown in Figure 7 is not consistent with the climate change trend shown in Fig 8(a). The gray scale curve based on polarized images of carbonate rock samples created in this study was mainly obtained using the gray scale formula. Since a given polarized image of a carbonate rock is different from the other images, the standard gray-scale formula may not be suitable for use with polarized images of carbonate rocks. For this reason, finite automata were used in this study [26].

### 2.3 Fitting of climate change curves

Curve fitting is a common operation in computer graphics. The curve established from the pore value of the carbonate rock will not have too many nodes due to the small number of carbonate rock samples. However, the number of curve nodes obtained using research methods such as stalagmites and lake sediments is very large. Thus, the number of nodes in the climate change curve established using the carbonate porosity should not be too small. It is not feasible to add curve nodes by appending carbonate samples. Curve fitting is a more feasible method. There are many curve fitting algorithms, and choosing an appropriate algorithm is a very important issue. The following points must be considered when selecting a curve fitting algorithm. The algorithm cannot change the general trend of the climate change curve, and the algorithm should provide sufficient fitting values. In this study, the cardioids curvy function was chosen to fit the climate change curve.

$$\begin{array}{c} \left\{ \begin{array}{c} x=a\times (2\times cos(t)-cos(2\times t))\\ y=a\times (2\times sin(t)-sin(2\times t))\\ t\in [0,360] \end{array}\right. \end{array}$$ (1)

**Fig 3:**
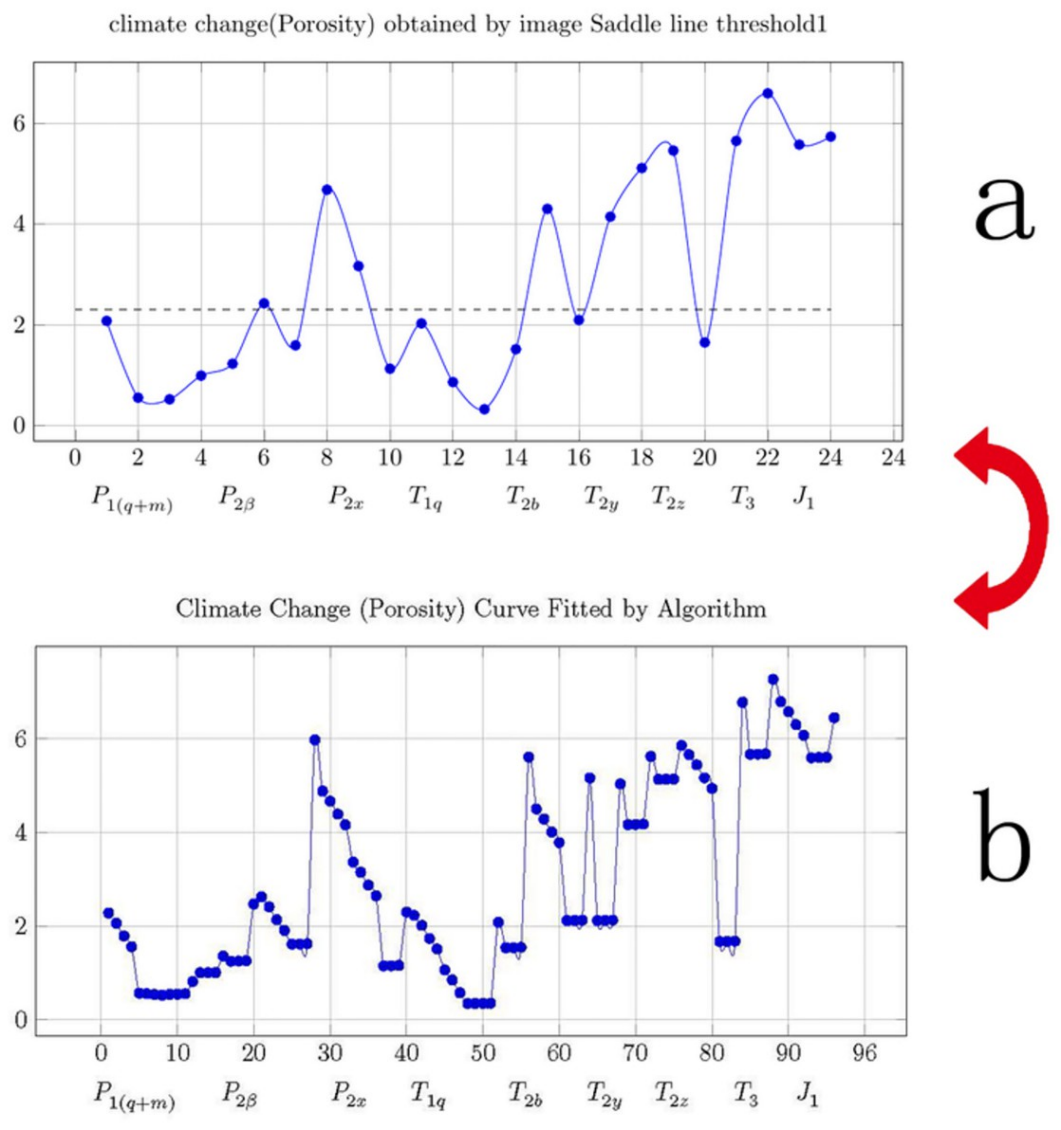
Fitting of climate change (porosity) curves.

According to the above curve fitting formula, the curve in Fig 8(b) was fit to that in Fig 3(b).

Fig 3a shows the climate change (porosity change) curve obtained using saddle line 1 in Table 1 (Column D); and Fig 3b shows the climate change (porosity) curve fitted using the cardioids curvy function.

**Table 1:** Porosity obtained from polarized light microscope images of carbonate rocks from the Jinping area using the saddle line threshold.

A	B	C	D	E	F	G	H	I	J
1-1	P 1(q+m)	135	2.078	135	0.21	135	0.216	135	0.001
1-2	P 1(q+m)	135	0.553	135	0.538	135	0.375	135	0.033
1-3	P 1(q+m)	135	0.521	135	0.863	135	0.876	135	0.014
2-1	P 2*β*	135	0.991	135	1.074	135	0.758	135	0.012
2-2	P 2*β*	135	1.228	135	1.54	135	1.6	135	0.017
2-3	P 2*β*	135	2.427	135	1.969	135	1.312	135	0.09
3-1	P 2x	135	1.593	135	1.645	135	0.046	135	0.353
3-2	P 2x	135	4.682	135	0.211	135	0.0008	135	0.312
4-1	T 1q	135	3.165	135	2.121	135	1.054	135	0.255
4-2	T 1q	135	1.129	135	1.623	135	1.154	135	0.028
5-1	T 2b	135	2.028	135	1.462	135	1.092	135	0.027
5-2	T 2b	135	0.863	135	0.704	135	0.798	135	0.001
5-3	T 2b	135	0.324	135	0.554	135	0.577	135	0.002
6-1	T 2y	135	1.516	135	1.626	135	0.159	135	0.238
6-2	T 2y	135	4.301	135	0.281	135	0.003	135	0.249
7-1	T 2z	135	2.096	135	0.848	135	0.03	135	0.35
7-2	T 2z	135	4.147	135	0.265	135	0.0034	135	0.364
7-3	T 2z	135	5.112	135	0.427	135	0.028	135	0.335
8-1	T 3	135	5.459	135	0.278	135	0.002	135	0.283
8-2	T 3	135	1.65	135	0.615	135	0.016	135	0.311
8-3	T 3	135	5.651	135	0.321	135	0.001	135	0.348
9-1	J 1	135	6.595	135	0.374	135	0.002	135	0.19
9-2	J 1	135	5.577	135	0.31	135	0.001	135	0.125
9-3	J 1	135	5.736	135	0.254	135	0.0009	135	0.188

A: serial number; B: Strata; C: Saddle line threshold 1; D: Porosity obtained using saddle line threshold 1 (%); E: Saddle line threshold 2; F: Porosity obtained using saddle line threshold 2 (%); G: Saddle line threshold 3; H: Porosity obtained using saddle line threshold 3 (%); I: Saddle line threshold 4; J: Porosity obtained using saddle line threshold 4 (%).

### 2.4 Optimal selection of image processing algorithms

The saddle line has been used for a long time as an image processing algorithm in the Jinping area. However, there are many curves in the saddle line curve family. Which curve is suitable for climate change research? Through repeated screening, it was found that the arithmetic mean of the carbonate porosity obtained from the following four curves was closest to the measured porosity value.

$$\begin{array}{c} \left[E_{n}\right]=\{\begin{array}{c} Z≐\left(x^{2}-y^{2}\right)/T_{1}\times T_{2}\times sin\left(\left(x+y\right)/256\right)\\ Z≐\left(x^{2}-y^{2}\right)\times T\times sin\left(\left(x+y\right)/256\right)\\ Z≐\left(-x^{2}-y^{2}\right)\times T\times sin\left(\left(x+y\right)/256\right)\\ Z≐\left(x^{2}-y^{2}\right)\times T\times cos\left(\left(x+y\right)/256\right) \end{array} \end{array}$$ (2)

**Fig 4:**
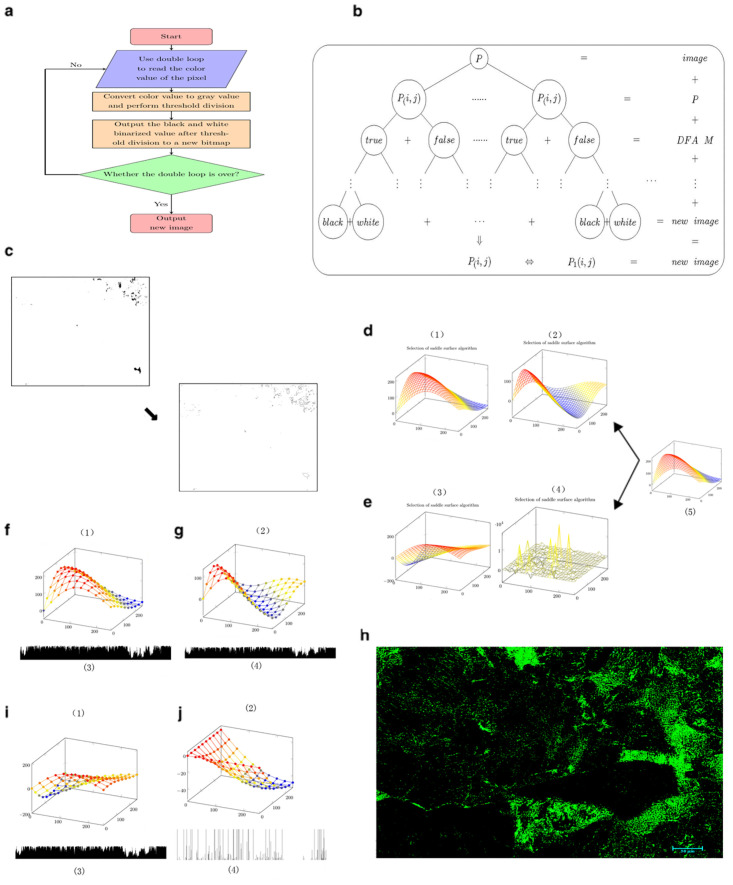
Optimization of image processing algorithms.

**Fig 5:**
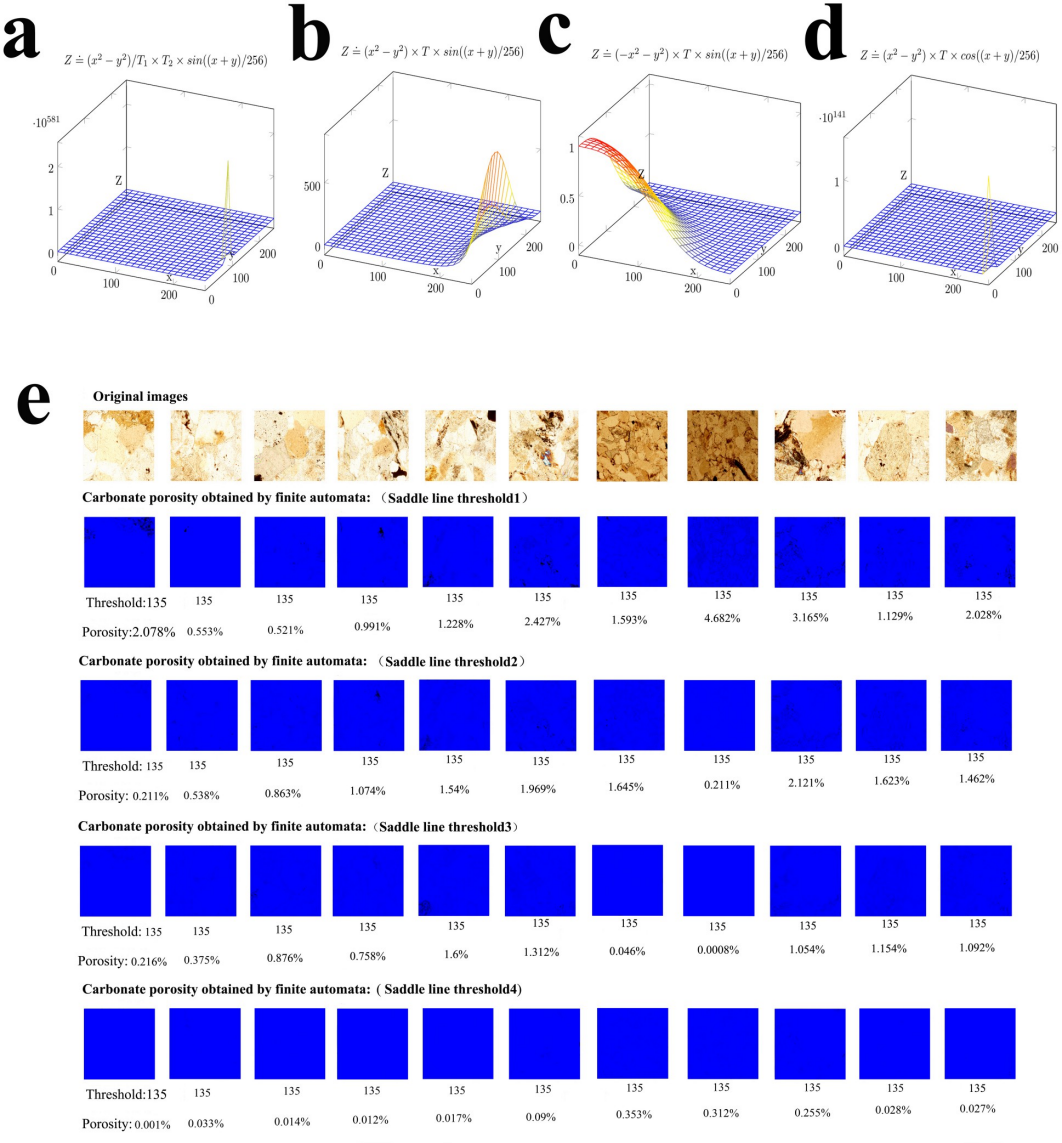
Saddle line mapping selection in finite automata.

All of the curves in Eq (2) were used as candidate curves in Fig 5, and further screening was performed (Fig 4).

**Fig 6:**
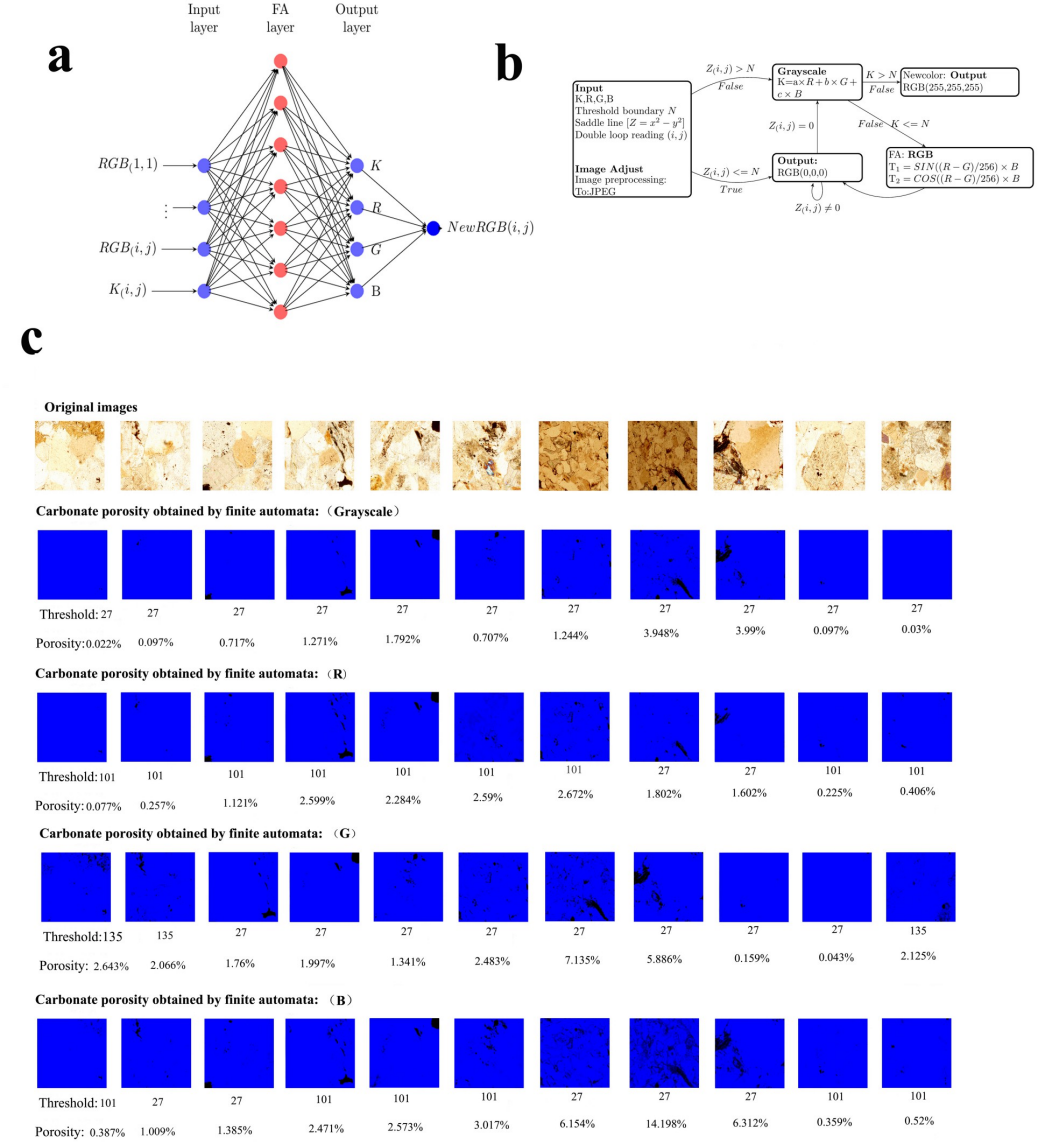
Applying finite automata to RGB values and grayscale values.

Fig 4a presents a flowchart of how the pixel point of a slide image is processed using an image processing algorithm; Fig 4b shows a schematic diagram of the implementation of the algorithm; and Fig 4c shows a binary image used to obtain a pore distribution map for imagej2x. Fig 4d(1), 4d(2), 4e(3) and 4e(4) show the alternative algorithm curves(four common saddle line mappings of Eq (2)). Fig 4f, 4g, 4i and 4j show the alternative algorithm curves(four common saddle line mappings of Eq (2)) and the corresponding R value curves. According to the R value curves obtained from the four saddle line mappings of Eq (2), the curve in Fig 4e(5) is the most suitable saddle line mapping for carbonate rock research. Thus, the curve in Fig 4e(5) was used in Fig 6. Fig 4h shows a new image obtained via gray-scale processing of the carbonate rock slide image using the curve in Fig 4e(5).

## 3 Results

### 3.1 Selection of saddle line mapping for the finite automata

Compared with the gray-scale curve, the algorithm for obtaining the porosity and pore size of a carbonate rock from a polarized image is slightly more complicated. In this study, the finite automata algorithm was used to analyze the polarized images of the carbonate rocks. The saddle line map was chosen as the map of the finite automata. There are four saddle-line maps that are often used in the Jinping area (Fig 5). The principle of the saddle line is to use the RGB value and gray value of the current pixel to generate a new RGB value and gray value using the saddle line formula. Then, the new RGB value and gray value are used to perform thresholding to obtain a black and white binary image. Based on this, the porosity of the carbonate rock is calculated using the black and white binary image. In this study, the four saddle-line maps were used to calculate the porosities from the polarized light microscope images of the carbonate rocks. Fig 5 shows the process of obtaining the porosity from polarized images of the carbonate rocks using saddle line mapping.

Fig 5a–5d show the four saddle-line maps; and e shows the original image and the carbonate porosity obtained from the RGB values or gray value using an automaton (saddle line mapping). The polarized light microscope images of all of the carbonate rocks were used to obtain their porosities using the method illustrated in Fig 5, and Table 1 was obtained. To distinguish it from the background color of the manuscript (white), the binary image is displayed in blue-black instead of white-black.

Fig 5 and Table 1 present the results of the porosity study of carbonate rock polarizing microscopic images using saddle line mapping. Based on these results, saddle line mapping is a good algorithm for porosity research involving carbonate image analysis. When saddle line mapping is applied to the finite automata, and different gray values or RGB values are used as the threshold of the finite automata, and the porosity of the carbonate rocks can be better obtained.

### 3.2 Applying finite automata to RGB values and grayscale values

After the finite automaton is determined, the finite automaton can be applied to the RGB values or grayscale values. In this study, the RGB values and grayscale values were used to calculate the porosities of the carbonate rocks from their polarized light microscope images. That is, the RGB value or gray value was processed according to the threshold value to obtain a black and white binary image, which was used to calculate the porosity of the carbonate rock. Fig 6 shows the process of obtaining the porosity from the polarized image of the carbonate rock using the RGB values and grayscale values.

Fig 6a shows the software architecture of the input, processing, and output of the RGB values and gray values in the finite automata; and b shows the data processing of the RGB values and gray values in the finite automata. The first row in Fig 6c shows the original image of the carbonate rock slide, and the other images in c are the binary images and porosity values obtained via thresholding using the gray value and RGB values. To distinguish it from the background color of the manuscript (white), the binary image is displayed in blue-black instead of white-black. The polarized light microscope images of all of the carbonate rocks were used to obtain the porosity using the method shown in Fig 6, and Table 2 was obtained.

**Table 2:** Porosity obtained from polarized light microscope images of carbonate rocks from the Jinping area using the RGB values and gray value.

A	B	C	D	E	F	G	H	I	J
1-1	P 1(q+m)	27	0.022	101	0.077	135	2.643	101	0.387
1-2	P 1(q+m)	27	0.097	101	0.257	135	2.066	27	1.009
1-3	P 1(q+m)	27	0.717	101	1.121	135	3.151	27	1.385
2-1	P 2*β*	27	1.271	101	2.599	27	1.76	101	2.471
2-2	P 2*β*	27	1.792	101	2.284	27	1.997	101	2.573
2-3	P 2*β*	27	0.707	101	2.59	27	1.341	101	3.017
3-1	P 2x	27	1.244	101	2.672	27	2.483	27	6.154
3-2	P 2x	27	3.948	27	1.802	27	7.135	27	14.198
4-1	T 1q	27	3.99	27	1.602	27	5.886	27	6.312
4-2	T 1q	27	0.097	101	0.225	27	0.159	101	0.359
5-1	T 2b	27	0.03	101	0.406	27	0.043	101	0.52
5-2	T 2b	122	0.716	135	0.224	135	2.125	27	0.669
5-3	T 2b	27	0.083	101	1.902	27	0.59	27	2.47
6-1	T 2y	175	5.908	27	8.047	27	8.429	27	13.92
6-2	T 2y	27	3.402	27	1.268	27	2.06	27	19.38
7-1	T 2z	175	1.878	27	6.69	27	7.697	27	22.93
7-2	T 2z	27	5.162	27	2.275	27	2.637	27	19.15
7-3	T 2z	27	5.039	27	2.897	27	3.235	27	19.37
8-1	T 3	27	3.31	27	1.502	27	5.639	27	12.72
8-2	T 3	27	4.217	27	2.43	27	6.141	27	11.008
8-3	T 3	27	3.318	27	1.912	27	6.055	27	15.556
9-1	J 1	27	1.858	27	0.95	27	3.765	27	13.722
9-2	J 1	27	2.04	27	0.523	27	5.043	27	15.803
9-3	J 1	27	6.62	27	4.01	27	4.802	27	22.476

A: serial number; B: Strata; C: Gray value threshold; D: Porosity obtained using gray value threshold (%); E: R threshold; F: Porosity obtained using R threshold (%); G: G threshold; H: Porosity obtained using G threshold (%); I: B threshold; J: Porosity obtained using B threshold (%).

Fig 6 and Table 2 present the results of the porosity study of carbonate rock polarizing microscopic images using finite automata. Based on these results, the finite automata algorithm is a good algorithm for porosity research involving carbonate image analysis. When studying the porosity of carbonate rock images, the finite automaton does not necessarily require the use of saddle line mapping to obtain the porosity of the carbonate rock. If different gray values or RGB values are used as the threshold of the finite automata, the porosity of carbonate rocks can also be obtained.

### 3.3 Use of image analysis results to generate climate change curves

**Fig 7:**
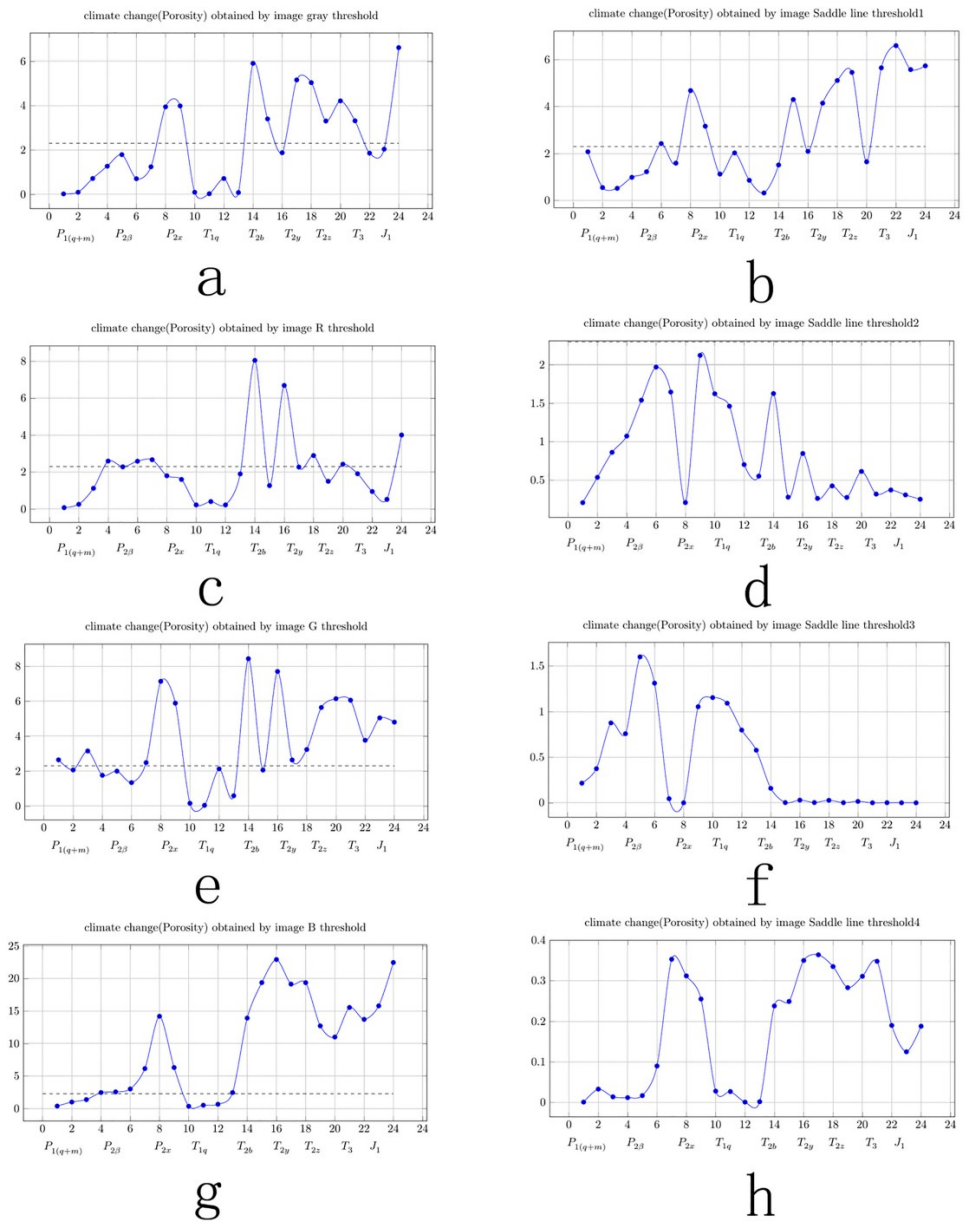
Climate change curves obtained using porosity changes.

Curves for loess and stalagmites can reflect climate change because loess and stalagmites are closely related to climate. The porosity of carbonate rocks is also closely related to climate change. The porosity curves of carbonate rocks should be similar to the curves for loess and stalagmites, which can reflect local climate changes. Therefore, in this study, it was assumed that the porosity curve created using the carbonate rock polarized images could be used as the climate change curve. When using polarized images of carbonate rocks for climate research, linking the results of the image analysis to the time axis is a difficult problem. If the time axis is not convincing, the climate change curve obtained will not be easy to believe or useful to the engineers. Since each rock sample is collected from a certain formation, the time axis of the climate change curve can be determined based on the formation age. Such a time axis is easy to understand and is readily accepted by engineers. Since the porosity of the carbonate rocks is related to the karst development rate, and the karst development rate is related to climate change, the porosity value of the carbonate rocks can be used to reflect the climate change. In this study, the carbonate porosity and pore size obtained from the image analysis were used as the climate change indicators. As is shown in Fig 7, when the porosity and pore size of the carbonate rocks were relatively large, as the climate was assumed to have been warm and humid. When the porosity and pore size of the carbonate rock were relatively small, as the climate was assumed to have been dry and cold.

Fig 7a shows the climate change (porosity change) curve obtained using the gray values in Table 2 (Column D). Fig 7c shows the climate change (porosity change) curve obtained using R values in Table 2 (Column F). Fig 7e shows the climate change (porosity change) curve obtained using G values in Table 2 (Column H). Fig 7g shows the climate change (porosity change) curve obtained using B values in Table 2 (Column J). Fig 7b shows the climate change (porosity change) curve obtained using saddle line 1 in Table 1 (Column D). Fig 7d shows the climate change (porosity change) curve obtained using saddle line 2 in Table 1 (Column F). Fig 7f shows the climate change (porosity change) curve obtained using saddle line 3 in Table 1 (Column H). Fig 7h shows the climate change (porosity change) curve obtained using saddle line 4 in Table 1 (Column J).

### 3.4 Using the existing data from published papers to obtain the climate change curve for the Jinping area

A great deal of climate research [27–78] has been conducted in western Sichuan, China, where the Jinping area is located. These studies provide a good comparative basis for climate change research in the Jinping area. However, some of the data in these papers are conflicting. To compare the existing data with the curve established from the porosity in this study, the results of the published papers on climate change in western Sichuan and adjacent areas were organized into a curve. This curve needs to reflect the trend of the climate change, not necessarily the value of the temperature at a certain point in time. Therefore, in this study, only the climate change curve was needed, not the temperature change curve. The climate change curve used in this study was the climate change trend curve. To facilitate the creation of the curves from the published data, the following principles of curve data collation were employed. Because the number of stalagmite studies in western Sichuan and the adjacent areas is relatively small, when the data were contradictory, the data were used in the order of loess, tree ring, sediment, and geomagnetism data. Data derived from historical documents were used when only historical document data and sediment data were available. In the event of conflicting data for the same region, the most recently published data were used. When data from different regions conflicted, the data from the region closer to western Sichuan were used. When of the results of loess or geomagnetic studies conducted in the same area conflicted, the most recently published results were used. When the climate change curves were constructed, the upper limit of the curve was set to 40 and the lower limit was set to 10. The baseline value for the climate change curve was set to 20. These values do not refer to temperature. The data points selected from the published articles were converted to stratigraphic ages first. The curve values for the research conducted using loess and sediments were adjusted according to the upper limit of 40 and the lower limit of 10. For the papers without climate change curves, the climate change values were determined based on the hot and cold descriptions in the paper. If the temperature was hot, the value was set as 35; if the temperature was warm, the value was set as 25; if the temperature was low, the value was set as 20; and if the temperature was cold, the value was set as 15. Even if the upper and lower temperature limits of the curve were reset, this would not affect the overall trend of the climate change curve. Therefore, in this study, the data from the published papers were used to organize the climate change curve and compare it with the porosity change curve.

**Fig 8:**
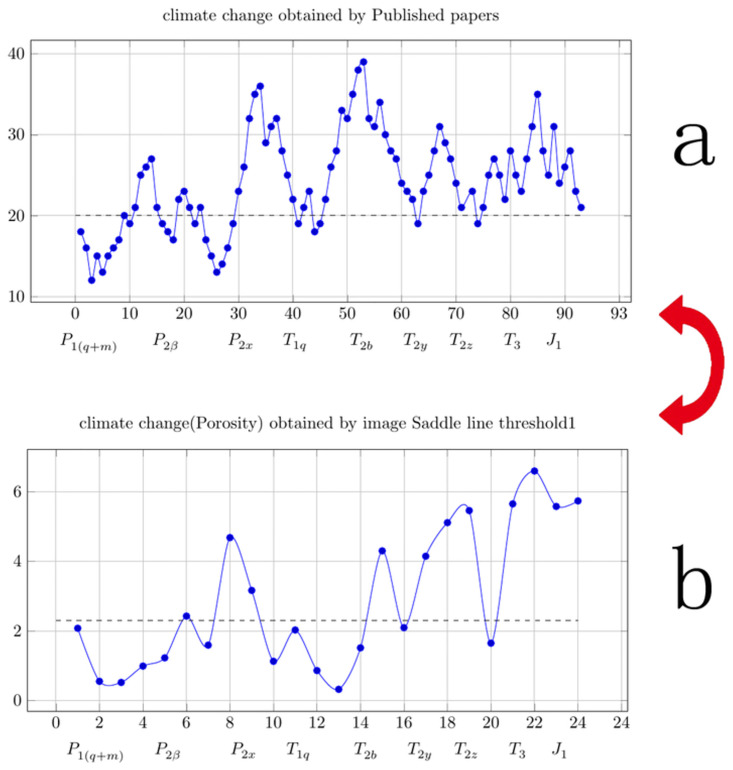
Using the existing data to obtain the climate change curve for the Jinping area.

Fig 8a shows the climate change trend map compiled using data from published papers; and Fig 8b shows the climate change (porosity change) curve obtained using saddle line 1 in Table 1 (Column D). It can be seen that the other curves are quite different from the climate change curve compiled using data from published studies.

## 4 Discussion and analysis

In climate change research conducted using carbonate stalagmites, the gray values of stalagmite profile images are often used to construct climate change curves. However, when using polarized light microscope images of carbonate rocks to conduct local climate change research, the use of grayscale images lacks the corresponding geological indicators. When using stalagmite profile images to conduct local climate change research, the use of gray values to construct climate change curves is supported by the corresponding geological indicators such as the stalagmite layers [79]. When using polarized light microscope images of carbonate rocks to conduct local climate change research, the gray value of the image may be affected by the polarized light microscope. Thus, in this study, a convincing parameter was needed to construct the climate change curve. The karst development rate and climate change are closely related in carbonate rock areas. The karst development rate and the porosity of carbonate rocks are also closely related. Therefore, it should be credible to use the porosity changes of carbonate rocks to establish climate change curves.

### 4.1 Comparison of the climate change trend obtained in this study with the climate change trend obtained using other means

The carbonate rock polarized microscope image curve obtained in this study is mainly the rock porosity curve, which is somewhat different from the published stalagmite climate change curve established using the gray value. Owing to the algorithm used to distinguish the differences, the curve established in this study was mainly established based on the rock image porosity, while stalagmite climate change curves are generally established using isotope chronology or standard image gray formula values. The principles of isotope chronology and image analysis are very different. The standard gray-scale formula for stalagmite images is also very different from the image processing algorithms used in this study. Therefore, in this study, the image standard gray-scale formula was not used to establish the climate change curve. The stalagmite samples used in several previous climate change studies were collected far away from western Sichuan, and it is doubtful whether the results obtained in these studies represent the climate change in western Sichuan.

### 4.2 Problems that should be paid attention to in establishing climate change curves using rock porosity

The following issues should be paid attention to when using polarized images of carbonate rocks to create porosity curves for use in climate change research. 1) The porosity obtained from a single polarized image of a carbonate rock via image analysis cannot be regarded as the value for the rock sample. Because the single polarized image only captures part of the rock slide. 2) The average rock porosity of multiple polarized images of a carbonate rock slide cannot be regarded as the value for the rock sample. At least these images should be divided into two groups, and the average rock porosity of each group should be calculated separately, and then, the two average values of the rock porosity of the two groups should be compared. If the average values of the rock porosities of the two groups are close, the current porosity identification algorithm is reliable. 3) The average rock porosity obtained via analysis of polarized images of carbonate rocks should be verified by measuring the porosity of the rock sample using the standard method. 4) If the average porosity obtained via analysis of polarized images of a carbonate rock is quite different from the measured porosity of the sample, the image analysis algorithm should be adjusted using the measured porosity of the sample as the target value. The average porosity obtained using the image analysis method should gradually approach the measured porosity of the sample.

### 4.3 Can this research method be used in other regions?

In other regions where regional climate change research needs to be conducted, the research method discussed in this study can be used as a reference. When applying the research method to regional climate change research in other regions, attention should be paid to the following premises. 1) There is no local climate research basis such as stalagmites and loess. 2) The research funds are not sufficient to conduct climate change research using stalagmites. 3) The local stratigraphic sequence is clear and detailed geological data are available. 4) The cost of obtaining polarized microscope images of local samples of carbonate rocks is not very high. 5) There is a wide distribution of carbonate rocks locally. As long as the above premises are met, it is feasible to apply the research method described in this paper to other regions.

## 5 Conclusions

In this study, through the analysis of carbonate rock images, the porosity change curve for the Jinping area was obtained. The climate change curve was obtained by collecting the research results of published papers on the Jinping area and its adjacent areas. Through comparison, it was found that the curve established using the saddle line algorithm for the carbonate image was the closest to the curve established using data from the published papers. In this study, the cardioids curvy function was used to fit the curve established using the saddle line algorithm for the carbonate images. The main conclusions of this study are as follows:

1. Published climate change data for Jinping and adjacent areas should be carefully identified when used. The differences in climate change curves obtained using different research methods in the same research area should be carefully analyzed.
2. Climate change will affect the karst development rate of carbonate rocks. Therefore, climate change can be analyzed by studying the porosity change of carbonate rocks. The change in the porosity of carbonate rocks can be used to study climate change in a carbonate rock area. The porosity change of carbonate rocks can be obtained via image analysis. The research on carbonate porosity changes based on image analysis can be used for climate change research in Jinping and adjacent areas.
3. The use of carbonate rock images in climate change research is based on the premise that the distribution characteristics of RGB values and gray values of carbonate rock images are suitable for image analysis karst research. Finite automata are a good model for image analysis of carbonate porosity. The saddle line algorithm is a good finite automaton algorithm.
4. When using carbonate rock images to study climate change, researchers may often encounter a small number of carbonate rock samples. The variation curve of the carbonate rock porosity obtained using the image analysis method can be fitted using a mathematical method.
5. The precondition of using carbonate rock images to study climate change is to obtain the porosity of carbonate rocksaccurately through image analysis technology. In the study of carbonate porosity, saddle line is an ideal image analysis algorithm. Before applying the saddle line algorithm to finite automata, the saddle line mapping must be carefully compared.
6. In the study of carbonate porosity, finite automaton is an ideal image analysis model. The threshold values of the gray value and RGB values used by the finite automaton must be carefully selected, and the setting of the threshold values directly affects the accuracy of the climate change curve.
7. In the study of porosity of carbonate rocks, the porosity change curve can be obtained using image analysis models, such as finite automata, to obtain the climate change curve of polarized micrographs of carbonate rocks. Polarized micrographs of carbonate rocks are a good basis for climate change research.
8. The cost of polarizedmicroscopy images of carbonate rocks is not high. Compared with other climate change studies, the research cost of climate change research using polarized light microscopic images of carbonate rocks is significantly lower. Therefore, the research method proposed in this paper can significantly reduce the cost of climate change research.
9. Compared with other climate change research methods, climate change trends can be satisfactorily verified based on the climate change trend obtained using polarized microscope images of carbonate rocks.
10. The gray value curve is often used as the climate change curve in the traditional study of climate change in carbonate rock areas. In this study, the RGB values of polarized microscope images of carbonate rocks were used to study the climate change trend using finite automata and saddle line mapping, which is an important innovation of this study. With the help of saddle line mapping, the B value of the polarizing microscope images of carbonate rocks plays an important role in the study of climate change trends using finite automata.
